# Remediation of Perceptual Deficits in Progressive Auditory Neuropathy: A Case Study

**DOI:** 10.3390/jcm13072127

**Published:** 2024-04-06

**Authors:** Gary Rance, Dani Tomlin, Eppie M. Yiu, Julien Zanin

**Affiliations:** 1 Department of Audiology and Speech Pathology, The University of Melbourne, Carlton, VIC 3053, Australia; dtomlin@unimelb.edu.au (D.T.); julien.zanin@unimelb.edu.au (J.Z.); 2 Department of Neurology, Royal Children’s Hospital, Parkville, VIC 3052, Australia; 3 Neurosciences Research, Murdoch Children’s Research Institute, Parkville, VIC 3052, Australia; 4 Department of Paediatrics, The University of Melbourne, Parkville, VIC 3052, Australia

**Keywords:** auditory neuropathy, Charcot–Marie–Tooth disease, axonal, auditory brainstem response, auditory processing, speech perception, remote-microphone listening device

## Abstract

Background: Auditory neuropathy (AN) is a hearing disorder that affects neural activity in the VIIIth cranial nerve and central auditory pathways. Progressive forms have been reported in a number of neurodegenerative diseases and may occur as a result of both the deafferentiation and desynchronisation of neuronal processes. The purpose of this study was to describe changes in auditory function over time in a patient with axonal neuropathy and to explore the effect of auditory intervention. Methods: We tracked auditory function in a child with progressive AN associated with Charcot–Marie–Tooth (Type 2C) disease, evaluating hearing levels, auditory-evoked potentials, and perceptual abilities over a 3-year period. Furthermore, we explored the effect of auditory intervention on everyday listening and neuroplastic development. Results: While sound detection thresholds remained constant throughout, both electrophysiologic and behavioural evidence suggested auditory neural degeneration over the course of the study. Auditory brainstem response amplitudes were reduced, and perception of auditory timing cues worsened over time. Functional hearing ability (speech perception in noise) also deteriorated through the first 1.5 years of study until the child was fitted with a “remote-microphone” listening device, which subsequently improved binaural processing and restored speech perception ability to normal levels. Conclusions: Despite the deterioration of auditory neural function consistent with peripheral axonopathy, sustained experience with the remote-microphone listening system appeared to produce neuroplastic changes, which improved the patient’s everyday listening ability—even when not wearing the device.

## 1. Introduction

Auditory neuropathy (AN) is a hearing disorder in which cochlear function is normal, but neural activity in the VIIIth cranial nerve and brainstem is disrupted. It is a relatively common condition affecting 1 in 7000 babies and approximately 1 in 400 special care nursery graduates [1,2]. Furthermore, AN cases account for 10% of all permanent hearing loss in childhood [3].

Progressive forms of AN also occur with a broad range of conditions, including genetic mutation, mitochondrial disorders, autoimmune diseases, metabolic disorders, and degenerative changes occurring as a consequence of noise trauma and aging [3,4].

Auditory neuropathy has been reported in a number of neurodegenerative diseases and may occur as a result of both the deafferentiation and desynchronisation of neuronal processes [3]. Deafferentiation involves a reduced number of activated nerve fibres and is commonly associated with axonal neuropathies, which deplete activity in the VIIIth nerve and central pathways without affecting the cochlear hair cells. The hereditary degenerative disorder Friedreich ataxia (FRDA) is the most well-described of these axonopathies and involves the loss of peripheral and cranial nerve fibres across multiple sensory and motor systems. The degree of auditory dysfunction in FRDA closely mirrors overall disease progression [3], and, as such, hearing assessments have been proposed as biomarkers to track the natural history of the disease and as outcome measures in intervention trials [5].

Some forms of AN may reflect the desynchronisation of neural activity due to demyelination. The loss of the myelin sheath results in the slowed or inconsistent propagation of neural activity and produces dyssynchrony when different fibres are demyelinated to differing degrees. This, in turn, affects the recording of auditory-evoked potentials and distorts the representation of complex auditory signals such as speech. An example of a demyelinating peripheral nerve disorder is Charcot–Marie–Tooth disease (Type 1), where affected patients present with delayed neural conduction in the auditory nerve/brainstem and varying degrees of perceptual disruption [6,7].

The perceptual symptoms of AN are distinct from those associated with peripheral (sensory) hearing loss, reflecting their different pathological mechanisms. Where perception in individuals with sensory loss is primarily limited by restricted sound detection and the impaired discrimination of frequency cues, patients with AN suffer the disruption of the neural code, which distorts the neural representation of acoustic timing cues. This, in turn, affects the ability to localise sound sources, discriminate rapidly changing sounds, and perceive signals in the presence of background noise [8,9,10].

The chief functional consequence of AN is impaired speech. Sensory loss can also disrupt speech perception, but the effects are typically less severe, and the degree of deficit is closely related to signal audibility. In patients with AN, the disruption of temporal processing (rather than audibility) is typically the limiting factor [9,10]. As a result, individuals with auditory neuropathy often report that speech is distorted and that they can ‘hear’ but not understand what is said to them.

In addition to experiencing signal distortion in optimal (quiet) conditions, individuals with AN may present with extreme difficulty in noisy situations. A major contributor to this deficit is impaired “spatial streaming”, which is the ability to differentiate a target signal from competing noise based on its location. This process affords individuals with normal auditory processing a 10 dB+ release from the masking effects of noise and is consistently disrupted by an auditory neural dysfunction [10].

The management of AN-related hearing deficits is challenging as neural distortion, rather than the ability to detect low-level sounds, is the primary limiting factor. As a consequence, conventional hearing aids, which make sounds louder but not necessarily clearer, are usually ineffective [8,11]. One intervention strategy that has had (limited) success in individuals with AN is the use of remote-microphone listening systems. These devices are specifically designed to improve the quality of speech signals in background noise and augment speech understanding not by making sounds louder but by improving the signal-to-noise ratio (SNR), i.e., the level of the target signal relative to the background noise. This is typically achieved through the use of a lapel-worn microphone, which digitally transmits the speaker’s voice directly to an ear-level receiver worn by the listener. Remote-microphone technologies have proven beneficial in other populations with auditory neural disruption, including children with Friedreich ataxia [12], neurofibromatosis [Type 1] [13], and autism spectrum disorder [14].

The purpose of this study is to describe the rapid and debilitating effects of hearing and communication changes that can occur in patients with neurodegenerative disease. In this case report, we track auditory function in a child with progressive axonal auditory neuropathy, evaluating hearing level and neural and perceptual changes over a 3-year period. Furthermore, we explore the effect of auditory intervention (remote microphone fitting) on everyday listening and neuroplastic development.

## 2. Detailed Case Description

We present longitudinal findings for a young male (Patient 1) with a history of congenital arthrogryposis and lower-limb predominant muscle weakness and areflexia. He underwent a number of surgical procedures for the correction of congenital talipes, hip dysplasia, and multi-level lower limb flexion contractures in the first few years of life. Neurologic examination at six years of age demonstrated marked lower limb wasting and distal more than proximal weakness. He had only a flicker of movement at the ankles and could flex his hips against gravity. He was able to walk with bilateral knee–ankle–foot orthoses and a walker. Lower limb reflexes were absent, and plantar responses were flexor. Upper limb reflexes were normal. Upper limb strength was relatively preserved, apart from some weakness around the shoulder girdle. A formal cognitive assessment at 7 years and 11 months showed overall average intellectual functioning, with relative strength in verbal skills compared to non-verbal and visuospatial abilities. His clinical course had been mildly progressive, with loss of ambulation at 9 years of age, driven by recurrence of lower limb flexion contractures.

Nerve conduction studies and electromyography performed at 5 and 7 years of age (and repeated at 10 years of age due to upper limb symptoms) were consistent with a motor greater than sensory motor axonal neuropathy (see Table 1).

Genetic testing using a Next-Generation Sequencing PathWest neuromuscular panel (PathWest Laboratories, Perth, Australia) identified a heterozygous *c.947G>A (p. R316>H)* pathogenic variant in exon 6 of the *TRPV4* gene which has previously been associated with Charcot–Marie–Tooth disease (Type 2C) and scapuloperoneal spinal muscular atrophy [15,16].

A range of behavioural and electrophysiologic auditory assessments was carried out at approximately 6-month intervals between the ages of 6 years 2 months, and 9 years 6 months.

**Table 1:** Nerve conduction results for Patient 1.

Nerve	4.5 Years of Age		7 Years of Age		10 Years of Age *	
	Amplitude	Conduction Velocity (m/s)	Amplitude	Conduction Velocity (m/s)	Amplitude	Conduction Velocity (m/s)
R median motor	**3.5** mV	73.9 m/sec	NP	NP	6.5 mV	NR
L median motor	NP	NP	5.0 mV	50.2	8.9 mV	83.0
R tibial motor	12.0 mV	61.7	12.5 mV	42.4	NP	NP
L tibial motor	NP	NP	NP	NP	17.1 mV	39.5
R peroneal motor	4.8 mV	72.4	9.2 mV	58.1	NP	NP
L peroneal motor	NP	NP	NP	NP	**0.03** mV	NR
R median sensory	**15.0 uV**	56.8	NP	NP	NP	NP
L median sensory	NP	NP	36.0 uV	50.3	**13.7 uV**	65.7
L ulnar sensory	NP	NP	NP	NP	**Absent**	**Absent**
L sural sensory	**5.8 uV**	78.7	NP	NP	17.9 uV	78.3
R sural sensory	39.9 uV	95.2	65.4 uV	40.3	NP	NP
EMG	Not performed		EMG of the right tibialis anterior and bilateral vastus lateralis muscles shows large and polyphasic motor unit potential. Fibrillations are present in the right vastus lateralis.		EMG of the left FDI and APB muscles showed reduced recruitment with large polyphasic motor unit potentials. No fibrillations were noted.	

* A further neurophysiologic study performed at 10 years of age due to cramping and fasciculations in the left upper limb. FDI = first dorsal interosseous, APB = abductor pollicis brevis, NP = not performed, NR = not recorded. Abnormal values are shown in bold type using a 5th percentile cut-off for motor and sensory amplitudes from Ryan et al. (2019) [17].

### 2.1. Sound Detection Levels

Behavioural hearing thresholds were determined for each ear to pure-tone acoustic stimuli at octave frequencies between 250 Hz and 8 kHz. On each test occasion, hearing threshold levels were within normal limits (≤15 dBHL) bilaterally.

### 2.2. Electrophysiology

**Figure 1:**
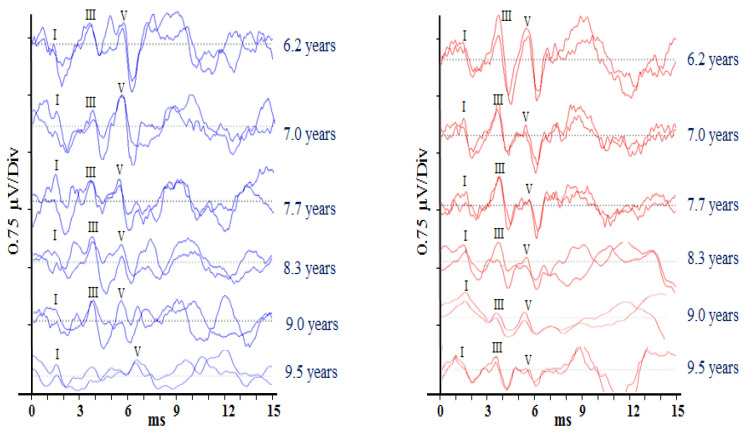
Auditory brainstem responses obtained from Patient 1 at (approximately) 6-month intervals from the age of 6 years 2 months to 9 years 6 months. Waveforms obtained for stimuli presented to the left ear are shown in blue, and to the right ear are presented in red. The roman numerals represent the positive peaks in the ABR waveform.

Auditory brainstem responses (ABRs) are scalp-recorded potentials reflecting synchronous neural activity from the auditory (VIIIth) nerve and brainstem. Differential recordings were made between the electrodes sited on the ipsilateral mastoid and vertex. A third electrode placed on the contralateral mastoid acted as a ground. Test stimuli were 100 µs acoustic clicks presented at a rate of 33.1 per second, and EEG activity following 2000 stimuli was averaged to produce each test run (Rance et al. [8]). Responses were analysed by two independent judges who determined the post-stimulus latency of ABR waves I, III, and V and the peak-to-peak amplitude for wave V. Averaged responses for each ear at each data collection point are presented in Figure 1. Wave V’s response amplitude decreased significantly over the recording period (*r* = −0.993, *p* < 0.001). Response latency (wave V), by contrast, showed no overall change (*r* = 0.612, *p* = 0.197), but a clinically significant (>0.3 ms) increase was observed for the left ear between the ninth year and 9 years and 5-month data points.

### 2.3. Auditory Temporal Processing

**Figure 2:**
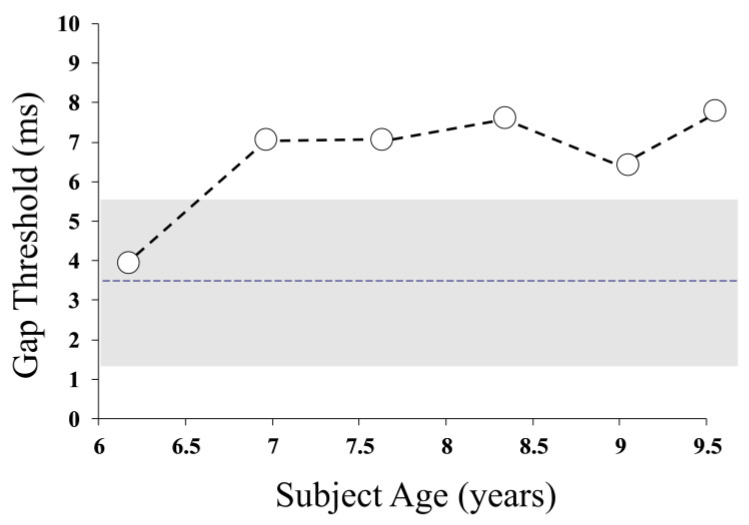
Auditory gap detection thresholds representing the minimum detectable silent period in a 500 Hz burst of noise presented to the left ear. The shaded area reflects the 95% performance range for normally developing children of equivalent age.

Auditory temporal resolution in Patient 1 was evaluated using a “gap detection” task, which sought the minimum noticeable silent period in a 500 ms noise burst [3]. The gap detection threshold worsened (from normal levels at baseline) over the study period, indicating that the patient’s auditory pathway was less able to encode brief changes in the acoustic signal (*r* = 0.814, *p* = 0.049) (Figure 2).

### 2.4. Speech Perception

Binaural speech perception in background noise was evaluated using the Listening in Spatialised Noise (LiSN-S) Test, which determines the subject’s ability to identify a target speech signal in the presence of competing speech noise [18]. The speech reception threshold (SRT) was established by varying the level of the target sentences relative to the noise to determine the minimum signal-to-noise ratio required to identify 50% of the words in the target sentences. SRT was determined in four test conditions that varied in terms of noise location (0° vs. 90° azimuth) and the vocal quality of the speaker used to produce the target and background signals (i.e., the same or different voice). The four stimulus conditions were DV90 (different voices spatially separated by 90°); SV90 (the same voice separated by 90°); DV0 (different voices from the same direction); and SV0 (the same voice from the same direction).

**Figure 3:**
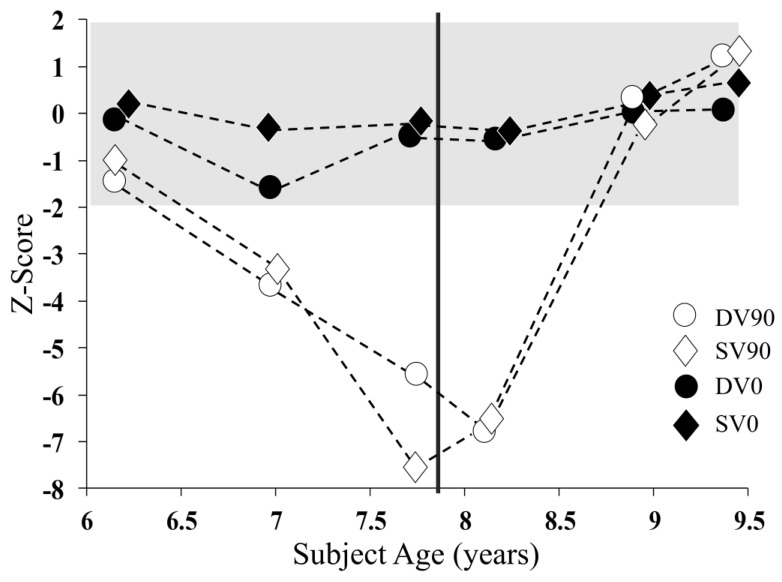
Binaural speech perception in noise (Listening in Spatialised Noise Test) results for Patient 1 over the 3-year study period. The data points are Z-scores indicating by how many standard deviations the participant’s results varied from the age-corrected mean. Findings for test conditions in which the target speech and noise emanated from different directions (DV90 and SV90) are represented by unfilled data points; conditions in which speech and noise were presented from the same direction (DV0 and SV0) are represented by filled points. The vertical marker represents the point at which the remote microphone listening system was provided.

Speech reception thresholds for test conditions not requiring the processing of binaural localisation cues (DV0 and SV0) were stable over the study period (Figure 3). In contrast, those conditions that involved the spatial separation of the target and background noise (DV90 and SV90) showed significant deterioration through the first 18 months of the study before recovering to normal levels following the provision of auditory intervention.

### 2.5. Device Fitting

Following teacher and parental reports of severe communication difficulties and academic delay, Patient 1 was provided with a personal remote-microphone listening system (Sonova AG, Zurich, Switzerland) at age 7 years and 7 months. This comprised a Phonak Roger-Focus receiver (worn behind the ear) by the child and a Phonak Roger-Touchscreen transmitter microphone worn at lapel-level by the child’s teacher. He responded well at fitting and subsequently maintained good device usage throughout the two-year study period, wearing the system 3–5 h per day.

Speech perception testing (at fitting) showed a significant perceptual advantage when wearing the device. Open-set speech perception (in background noise) was assessed in the free field as per Rance et al. [12] and showed a significant device advantage, with the patient’s discrimination score improving from 38% in the unaided condition to 78% when wearing the remote-microphone system. That is, an improvement occurred when the patient was able to hear twice as much of the augmented speech signal when device-aided.

**Figure 4:**
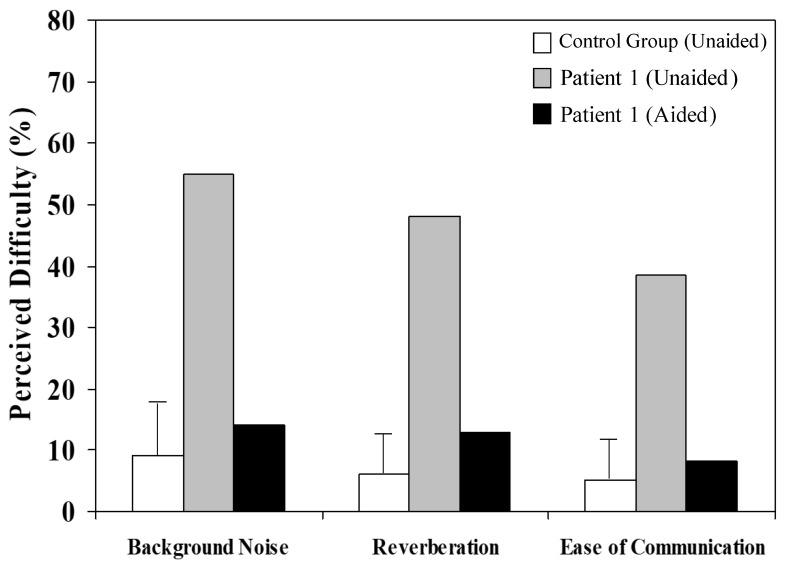
Abbreviated profile of hearing aid benefit (APHAB) results reflecting everyday listening and communication ability for Patient 1 in unaided (grey bars) and remote-microphone-aided (black bars) conditions. The white bars show mean ± 2SE ranges for normally developing children of a similar age [12].

In addition to showing perceptual improvements in formal (laboratory-based) testing, Patient 1 considered that his hearing and general communication were improved when wearing the remote microphone listening system. After 4 weeks of device experience, he undertook a hearing disability questionnaire (Abbreviated Profile of Hearing Aid Benefit [APHAB]) and rated his “perceived difficulty”—the proportion of everyday situations in which he experienced a listening difficulty—to be substantially lower when device-aided (Figure 4).

## 3. Discussion

This case report is the first to describe the mechanism (progressive axonopathy) underlying auditory dysfunction in TPRV4-linked Charcot–Marie–Tooth disease (type 2C). Furthermore, it is the first to demonstrate neuroplastic auditory pathway changes following intervention in a child with auditory neuropathy.

Charcot–Marie–Tooth disease (CMT) affects about 1 in 3000 children and presents with symptoms reflecting peripheral neuropathy, including orthopaedic abnormality, muscle atrophy/weakness, and sensory disruption. The disease is divided into demyelinating conditions (e.g., CMT type 1 [dominantly inherited] and CMT type 4 [recessively inherited]), axonal forms (including CMT type 2, typically dominantly inherited), and intermediate forms, such as those with X-linked inheritance. Auditory neuropathy is a commonly reported feature in both axonal and demyelinating sub-types [6,19].

While impaired sound detection has been reported in both adults [20] and children [21] with TPR4-linked CMT2C, no previous studies have explored the mechanisms underlying auditory dysfunction in these patient groups. Recent cross-sectional studies involving CMT patients with other forms of CMT2 (i.e., axonal) pathology have, however, consistently reported reduced or absent brainstem potential, perceptual deficits out-of-proportion with sound detection ability [22,23], and degrees of auditory dysfunction correlated with overall disease progress [22].

In the current CMT2C case, we found a progressive ABR amplitude reduction over the 3-year study period. There are two mechanisms that might underpin this degeneration. The first is axonopathy. Histopathological evidence from the temporal bones of an individual with CMT2 has indicated a selective loss of auditory ganglion cells and nerve fibres [24]. The loss of these neural elements in Patient 1 may have resulted in a deafferentiation—or decrease in the overall amount of activity available to contribute to the averaged evoked response and, hence, reduced ABR amplitudes. The second possible pathologic mechanism is dyssynchrony. The amplitude of evoked potentials is also affected by the temporal precision of neural activity. Transient responses (such as the ABR) are typically extracted from the EEG via an algebraic summation process. This technique requires that the timing of the neural discharges after each stimulus be almost identical, and work by Starr and colleagues has indicated that temporal variations as little as 0.5 ms may make an averaged response that is unrecognisable within the background EEG [25]. Dyssynchronous neural activity may be occurring in this case as a secondary consequence of axonopathy. The loss of VIIIth nerve fibres produces aberrant neural firing patterns when variable conduction speeds occur in damaged axons [26]. Secondary demyelination may also occur as a result of axonal damage and can affect the efficiency and consistency of neural activity when different fibres are affected to different degrees. Demyelination may also affect the latency and morphology of the evoked response and, as such, could explain the ABR waveform changes observed for the left ear in Patient 1 through the latter stages of the trial.

Consistent with the progressive neural abnormality reflected in the evoked potential findings, Patient 1 showed evidence of worsening auditory temporal resolution over the course of the study. His ability to identify brief changes (silent periods) in an acoustic signal was significantly poorer at the study’s end when he required a gap duration approximately twice that needed at the baseline to recognise the silent period. Changes in this order are functionally significant, as running speech involves a rapidly changing acoustic signal requiring the discrimination of features lasting only 10s of milliseconds to differentiate between speech sounds [9].

Despite the fact that there was a clear deterioration in Patient 1′s auditory neural function and temporal processing, he showed no discernible change in overall disease severity throughout the study period. This is somewhat surprising as auditory pathway changes usually occur in concert with overall disease progress [3,22]. There is, however, evidence that the auditory pathway is more sensitive to subtle neuropathic change than other sensory or motor systems. For example, functional hearing (speech perception) changes often present years before other neural symptoms in diseases involving generalised neurodegeneration [8]. As such, the observed auditory changes may have been a reflection of (otherwise) sub-clinical disease progression.

Binaural speech perception in noise showed a dramatic deterioration over the first 1.5 years of study (Age 6.2–7.7 years). A concept that was particularly affected was discrimination, requiring the perception of inter-aural acoustic cues (i.e., signal level differences of around 5–15 dB and timing differences of around 50 µs between ears). In normal listeners, these subtle cues provide spatial (sound direction) information that can spatially separate a target signal from the background to optimise perception [27]. The disruption of binaural speech perception has been reported previously in conditions that affect the consistency of auditory input (e.g., fluctuating conductive hearing loss [28]) and in patients with demyelinating or axonal neuropathies [10].

The provision of auditory intervention (a remote microphone listening system) resulted in a significant perceptual benefit. When wearing the device, Patient 1′s perception of speech in background noise improved to within normal limits. That is, his aided perceptual ability was equivalent to that of an unaided, normally developing child [13]. This level of improvement is consistent with that reported previously for patients with progressive axonal neuropathy [12]. Furthermore, his binaural perception (spatial processing ability) improved throughout the trial period, even when not wearing the device (Figure 3).

Clearly, sustained exposure to the acoustically enhanced signal provided by the device resulted in some form of neuroplastic change, but the mechanism is unclear. Binaural interaction, the physiological component of spatial listening, occurs in the auditory brainstem, where inputs from the left and right ears are combined and compared. The improved consistency of neural firing in this region (superior olivary complex, lateral lemniscus, and inferior colliculus) has been demonstrated in some populations with auditory neural deficit (notably children with dyslexia) with the provision of auditory training [29] and with remote-microphone device fitting [30]. In Patient 1, however, neurodegeneration in the auditory brainstem (and the associated disruption of auditory processing), in fact, progressed through the intervention period, suggesting that the observed speech-in-noise improvements were the result of changes elsewhere in the auditory pathway.

One possibility is that attention-linked processes at the level of the auditory cortex may be involved. There is increasing evidence of the strengthening of auditory attention skills in children following a period of remote microphone use [31,32]. Binaural interaction occurs in the lower brainstem, but spatial listening is thought to include a top-down hierarchical prediction at the cortical level, which is compared to the incoming signal. Any discrepancy between the signal and the prediction is transmitted back up to the higher level. This process is an attention-linked mechanism. With a degraded signal (which would occur in this case of neural distortion), a recalibration of higher-level representation occurs. As hypothesised by Koohi et al. [33], who found an improvement in spatial listening in stroke patients after a period of remote microphone use, attention-optimising synaptic gain may occur. This improves the precision of the auditory cortex in the hierarchical two-way signal-matching process. Thus, the compensatory strengthening of attention skills, initially primed by the use of the remote microphone system, may support neuroplastic changes in the higher auditory pathways, resulting in improved binaural processing and speech perception of noise.

### Study Limitations

This work has limitations. As always, with single case studies, the conclusions need to be interpreted with caution. Furthermore, while the findings for Patient 1 were either compared with published normative data or with himself over time, they were not considered in relation to a matched control. Future studies might include larger participant numbers and a cohort of age, gender, and hearing-level-matched children with no known neurological abnormalities.

## 4. Conclusions

In summary, this case study presents the first longitudinal auditory evaluation of a child with Charcot–Marie–Tooth (Type 2C) disease, tracking the progressive deterioration of auditory neural activity and concomitant loss of functional hearing ability. We provide evidence that the provision of a remote microphone listening system can result in improved perception and everyday communication. Furthermore, we demonstrate, for the first time in a patient with auditory neuropathy, that sustained experience with the augmented signal may produce neuroplastic changes that improve functional hearing ability—even when the child is not wearing the device.

## Data Availability

All data underlying the results are available as part of the article and no additional source materials are required.
